# The Impact of Ken Warren’s Leadership of the Rockefeller Foundation’s Great Neglected Diseases Program on the Future of Malaria Research

**DOI:** 10.4269/ajtmh.17-0849

**Published:** 2017-12-06

**Authors:** Louis H. Miller

**Affiliations:** National Institute of Allergy and Infectious Diseases, National Institutes of Health, Rockville, Maryland

In his recent book, Conrad Keating tells the story of Ken Warren’s tenure as the Director of the Rockefeller Foundation (RF) Great Neglected Diseases (GND) Program from 1977 to 1987. In 1977, when Ken Warren’s story at the RF begins, the Foundation supported two major programs in the developing world: one in agriculture (for which Norman Borlaug was awarded the Nobel Peace Prize in 1970 for the Green Revolution) and one in health. John Knowles, the Director of the RF from 1972 to 1979, had the ambition to increase the Foundation’s impact on world health, and for that, he brought Ken Warren to head the Health Division. Warren believed that this could best be accomplished through the support of basic research focused on diseases that plagued the developing world. Thus, Warren created the GND Program. His strategy to build a strong basic research component in the GND Program was to engage top leaders in science from around the world. Conrad Keating describes Ken Warren’s many achievements at the RF during the years he was head of the GND Program. Keating ends his book with a chapter entitled “The Fall,” describing Warren’s exit from the RF, leaving the impression that Ken’s legacy to the GND Program also ended with his departure. What seems missing in Keating’s telling of the story is the enormous impact Warren had on building basic research in diseases of the developing world, particularly in malaria, that continues to the present day. I entitle this discussion “Beyond the Fall.” I will comment on the contributions of four individuals that Ken Warren brought into the GND Program: David Weatherall at Oxford University; Gus Nossal at the Walter and Eliza Hall Institute in Melbourne, Australia; and Hans Wigzell and Peter Perlmann in Sweden.

Science is a slow process, with progress measured in decades, not years, and success requires long and often circuitous routes of discovery. The first major advance in malaria research was the discovery of quinine, an antimalarial that is still effective today, and this advance led to the development of synthetic derivatives of quinine, beginning with Ehrlich’s work in the early 1900s, and eventually to 4-aminoquinolines, derivatives of which are still used today. The discovery of dichloro diphenyl trichloroethane as a potent insecticide for the mosquito vector of the malaria parasite was a fortuitous one that contributed to the elimination of malaria in Italy and many other countries. The discovery of the antimalarial properties of herbal artemisinin dates back to recipes written 1,700 years ago by Ge Hong in China. Artemisinin was rediscovered by Tu Youyou in 1971 while working on Mao’s antimalarial program in support of the Vietnamese in the Vietnam War, in which malaria was a greater threat to soldiers than the US army. Ironically, artemisinin’s vital impact on the treatment and control of malaria continues throughout the world, especially in Africa. Where the next breakthrough in the fight against malaria will come depends on the unpredictability of science.

Ken Warren believed that investments in basic science would lead the way in combating malaria in the developing world; and to that end, he engaged top research scientists around the world to participate in the GND Program. I begin with David Weatherall, who described in the foreword to Keating’s book the first GND Program meeting to which Ken Warren invited a number of distinguished scientists. It was at this meeting that Peter Williams, the director of the Wellcome Trust, invited David to his room to discuss the future of Medicine in the Tropics in the UK, a program that David built at Oxford to make the university a world leader in malaria research. Through this program, Oxford supported several excellent malaria research groups led by outstanding young scientists including Kevin Marsh in Kenya, Nick White in Thailand, and Adrian Hill and Dominic Kwiakowski at Oxford, all of whom continue to lead the most influential malaria research programs in the world. We will never know the true influence of the GND Program meeting on Weatherall and the Oxford community, but I suspect it was great.

Equally important was Gus Nossal, another participant at the first GND Program meeting, who was the director of the Walter & Eliza Institute. Gus first recruited David Kemp, a leading molecular biologist, and soon added two of David’s students, Graham Brown and Alan Cowman, and then Alan’s colleague, Brendan Crabb, who is now Director of the Burnet Institute. Cowman now leads a large group of malaria scientists studying all aspects of malaria, including work in Papua New Guinea under Ivo Mueller. Gus also sent one of his bright students, Michael Good, to train with me at the National Institutes of Health. Michael returned to Australia and now leads a group working on malaria vaccines.

In Sweden, malaria research was led by Hans Wigzell and Peter Perlmann whose student, Mats Wahlgren, leads the work at the Karolinska Institute.

In rating Ken Warren’s impact on malaria research, I look to the large diaspora of malaria researchers that I trace back to the original meeting of the GND Program in 1978 at the Rockefeller University in New York and that now lead malaria research in all parts of the world. I am sure that research by these distinguished scientists and their trainees will have a tremendous future impact on easing the toll of this terrible disease on the world’s most vulnerable people.

